# Comparison of methods for genomic localization of gene trap sequences

**DOI:** 10.1186/1471-2164-7-236

**Published:** 2006-09-18

**Authors:** Courtney A Harper, Conrad C Huang, Doug Stryke, Michiko Kawamoto, Thomas E Ferrin, Patricia C Babbitt

**Affiliations:** 1 Department of Biopharmaceutical Sciences, University of California San Francisco, 1700 4th Street, San Francisco, CA 94143-2250, USA; 2 Department of Pharmaceutical Chemistry, University of California San Francisco, 1700 4th Street, San Francisco, CA 94143-2250, USA

## Abstract

**Background:**

Gene knockouts in a model organism such as mouse provide a valuable resource for the study of basic biology and human disease. Determining which gene has been inactivated by an untargeted gene trapping event poses a challenging annotation problem because gene trap sequence tags, which represent sequence near the vector insertion site of a trapped gene, are typically short and often contain unresolved residues. To understand better the localization of these sequences on the mouse genome, we compared stand-alone versions of the alignment programs BLAT, SSAHA, and MegaBLAST. A set of 3,369 sequence tags was aligned to build 34 of the mouse genome using default parameters for each algorithm. Known genome coordinates for the cognate set of full-length genes (1,659 sequences) were used to evaluate localization results.

**Results:**

In general, all three programs performed well in terms of localizing sequences to a general region of the genome, with only relatively subtle errors identified for a small proportion of the sequence tags. However, large differences in performance were noted with regard to correctly identifying exon boundaries. BLAT correctly identified the vast majority of exon boundaries, while SSAHA and MegaBLAST missed the majority of exon boundaries. SSAHA consistently reported the fewest false positives and is the fastest algorithm. MegaBLAST was comparable to BLAT in speed, but was the most susceptible to localizing sequence tags incorrectly to pseudogenes.

**Conclusion:**

The differences in performance for sequence tags and full-length reference sequences were surprisingly small. Characteristic variations in localization results for each program were noted that affect the localization of sequence at exon boundaries, in particular.

## Background

High-throughput gene interruption projects have greatly increased the number of loss-of-function knockout genes available for study [1]. Correct identification of these genes provides a necessary foundation for their use for biomedical discovery, including minimizing the number of time-consuming phenotype experiments that need to be undertaken. Until recently, interrupted knockout genes have been identified primarily using the alignment program BLAST [2] to match gene trap sequence tags, which represent the region of an interrupted gene near the site of disruption, with gene transcripts. While transcript identification can generally provide high confidence gene annotation information for over 75% of such knockouts [3], transcript databases do not provide full coverage of the genome, limiting the number of genes that can be identified. Redundancy in transcript databases also makes it difficult to obtain a unique identification for sequence tags, which are relatively short.

Sequence quality can also be an issue with gene trap sequence tags, since the prevalent method of generating these tags often results in relatively low-quality sequence. BayGenomics [3] and other members of the International Gene Trap Consortium (IGTC) [4,5] typically use 5' RACE [6], a common method for amplifying sequence from gene insertion events. This method generates sequence from only one strand of DNA, and often generates only relatively short sequences, with sequencing errors accumulating especially towards the 3' end. To obtain sequence tags that are sufficiently long to uniquely identify most genes, BayGenomics, for example, uses a limit for the acceptable quality of a base call that is lower than the generally accepted threshold (a Phred [7] minimum score of 14.6 rather than the default score of 30) [3]. The consequence of using such a low threshold is that nucleotides are assigned incorrectly somewhat more often than with the default threshold value. This problem can interfere with annotation. [see Additional file 1 for an example.] Additionally, sequence tags generated by 5' RACE occasionally have non-templated nucleotides at their termini [8]. In one large-scale 5' RACE experiment, only 57% of clones generated sequences that were sufficiently long and unambiguous to be identified by alignment with a gene transcript [9].

As curation of the mouse genome has improved, direct localization has become the strategy of choice for associating sequence tags with specific genes. This has an advantage in minimizing imprecise and confusing annotations arising from redundancy in mRNA databases. Moreover, this approach reflects the biological reality of the insertion of a reporter gene into genomic sequence and provides a more context-based view of the gene by associating it with the many types of information available at the genome browser Web sites.

The choice of alignment program is a major consideration in localizing sequences on the genome. BLAST, which was developed for comparison of evolutionarily diverged sequences, is prohibitively slow in this application. Several newer algorithms have been developed to rapidly align nearly identical sequences. Implementations in common use are MegaBLAST [10], the Sequence Search and Alignment by Hashing Algorithm (SSAHA) [11], and the BLAST-like Alignment Tool (BLAT) [12]. Each is currently in use at one of the primary genome browser sites and, in addition, each is available for stand-alone use. MegaBLAST is used at the National Center for Biotechnology Information (NCBI) [13], SSAHA is used at Ensembl [14], and BLAT is used at the University of California Santa Cruz (UCSC) [15]. While all of these algorithms have been individually benchmarked for the genome browsers with which they are used, their performance with sequence tags has not been established, nor have the results from the stand-alone versions of these programs been compared with the gene annotations available at the genome browser sites. Establishing the effect of low quality and short sequence length on gene localization protocols is beneficial to research groups that work with gene tag and similar sequences, including other types of expressed sequence tags (ESTs) or genomic tags.

MegaBLAST is similar to BLAST in that it splits a query sequence into non-overlapping fragments and searches for exact matches to the genome to find the regions of highest identity. These perfect matches are then expanded to align the longest region of significant similarity. MegaBLAST uses a greedy algorithm that incorporates simplified gap and insertion/deletion penalties relative to BLAST and limits the number of alignments to be explored in extending the alignment beyond a perfect match seed. These alterations are justified because of the high levels of similarity expected between query and database sequences and the expectation that the alignment will not contain many mismatches or gaps. For sequences with greater than 97% identity, MegaBLAST is an order of magnitude faster than BLAST without any loss of alignment accuracy [10].

SSAHA uses a different approach to take advantage of the high similarity expected between a query sequence and the genome. An index of all non-overlapping fragments of a set length (*k*) is created from the genome sequence and stored with the associated positions. The query sequence and its reverse complement are broken into all possible fragments of length *k*, including overlapping fragments, and compared with the genome index to identify exact matches. Matches are sorted to find contiguous matching segments that are reported if they exceed a threshold, set by default to 2*k*. SSAHA is extremely fast, but due to the need to store the genome index and fragment locations, has relatively large memory requirements.

BLAT uses a multi-stage algorithm which searches for regions of similarity, aligns those regions, aggregates aligned regions in close proximity, and adjusts the boundaries of aligned regions to correspond with canonical splice sites. The initial search stage operates in a manner very similar to SSAHA. The genome database is broken into non-overlapping fragments of length *k*, then all *k*-length fragments of the query sequence and its reverse complement are associated with matching locations in the genome. The matches are sorted and grouped by proximity and those regions of the genome with a minimum of 2*k *contiguous matches are aligned with the query sequence. The alignment stage extends matching regions as far as possible, merges overlapping matches, links matches that fall in order on the genome into a single alignment, and fills in regions of the alignment corresponding to gaps of identical length in the query and genome sequences. Positions of gaps in the alignment, which may correspond to introns, are matched to the consensus splice site GT/AG whenever possible.

The work reported here provides a comparison of the performance of the stand-alone versions of SSAHA, MegaBLAST, and BLAT for a set of mouse gene trap sequence tags. The sequence tags were generated through untargeted gene-trap experiments, which detect instances where the insertion vector interrupts an intron of a gene expressed in embryonic stem cells [1]. As the genome coordinates of our sequence tags are not known, the localizations of their cognate genes were used as a proxy. These genes were identified by using the BLAST program to align the sequence tags with gene transcripts (see Methods for details).

The genome coordinates of many genes in the mouse genome are defined differently depending on which genome browser site provides the information. This is because each browser uses a different combination of localization programs, sequence analysis tools, and manual curation to arrive at their final annotations. Additionally, the localization program used in the annotation protocol may differ from the localization program provided to users of the genome browser. For example, Ensembl uses the exonerate program [16] to generate localization coordinates reported at their site. However, when a user seeks to localize a gene at the Ensembl site, the SSAHA algorithm is used to perform that task. This differs from NCBI and UCSC, where the localization algorithms used to generate annotations for the genome, MegaBLAST and BLAT respectively, are also used by the genome browser to localize sequences input by users. In order to provide a fair comparison between the algorithms, only sequence tags matched with genes having exactly the same coordinates at Ensembl, NCBI, and UCSC were used in this study. To determine whether errors in the localization of sequence tags using the stand-alone versions of these programs was due to the nature of the sequence tags themselves or to differences in how the stand-alone programs perform relative to the protocol in which they are used to localize full-length genes at each browser site, we also localized the set of gene transcripts matched with sequence tags as a control. Our sequence set consisted of 3369 sequence tags associated with 1659 genes with uniformly assigned coordinates on the mouse genome.

## Results and discussion

**Figure 1:**
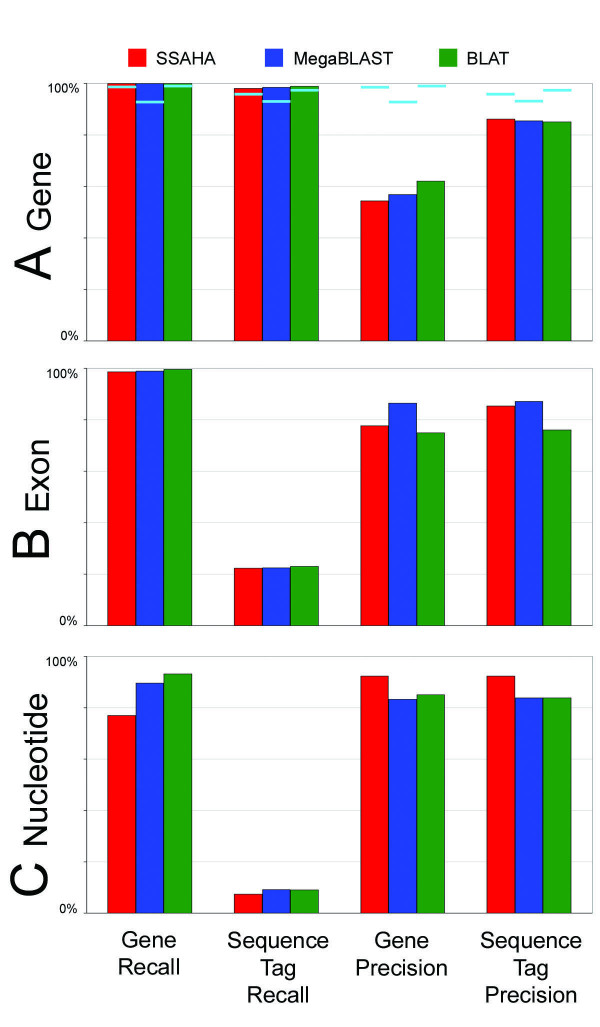
**Recall and precision for each localization algorithm**. Results for SSAHA are shown in red, MegaBLAST in blue, and BLAT in green. The first column represents the recall obtained with full-length gene query sequences. The second column shows the recall obtained with sequence tag queries. The third and fourth columns display the precision of each algorithm when used to localize full-length genes and sequence tags, respectively. (A) Recall and precision at the level of the gene, as measured by overlap of at least one nucleotide between a set of localizations by an algorithm and the region of the genome containing the gene. Cyan lines indicate the recall and precision achieved when only the top hit is considered. (B) Exon recall and precision, as measured by an overlap of at least one nucleotide between the known localization of an exon and a match. Sequence tags are shorter than full-length genes and therefore typically contain sufficient sequence information to match only a few exons of any gene, leading to low recall at the exon and nucleotide levels. This does not indicate failure by the localization programs. (C) Nucleotide recall and precision, as measured by a match between a nucleotide in the known localization of a gene and a nucleotide from a query sequence localization.

Our results show differences in the localization performance with respect to recall and precision at each of three levels of granularity investigated, gene, exon, and nucleotide (Figure 1). The recall score indicates the percentage of true positives that were detected. Precision indicates the percentage of matches reported which correspond to true positives.

### Localization to the correct gene

With respect to recall, our study shows that researchers who wish to link a sequence with information associated with the genome may confidently use any of the three localization programs considered in this study. SSAHA, MegaBLAST, and BLAT successfully localize each of the 1659 full-length genes in the test set to a genomic region that fully or partially matches the known coordinates of the corresponding gene (Figure 1A). Sequence tags fare nearly as well, with all programs reporting localization to the correct region of the genome for >98% of the 3369 sequence tags used in this study.

Repeat-masking of the genome accounts for the majority of the small number of failures in localizing sequence tags to the correct genes. Online localization is performed against masked genomic sequence by default as this ensures that results are returned quickly and that relatively few correct localizations are missed, despite the fact that as much as 50% of the genome consists of repeated elements [17]. In this study, less than 2% of sequence tags in the test set returned no localization results with one or more programs because they overlap fully or partially with regions removed by masking. Additionally, five sequence tags that localize to repeat regions have erroneous matches that exceed the minimum score required by each program, and so are localized incorrectly. In contrast, use of an unmasked version of the genome results in 100% recall for the test set of sequence tags, but increases the number of incorrect localizations by as much as ten-fold. Moreover, using an unmasked version of the genome increases computation time substantially (Table 1).

**Table 1:** Computation times in seconds for each algorithm.

		Computation Time in seconds		
	
	# of Sequences	MegaBLAST	SSAHA^a^	BLAT^a^
Full-length Genes	3320	1767 (40578)^b^	361 (29895)	1434 (204331)
Sequence Tags	7043	223 (1025)	38 (5806)	276 (854)

^a ^Reported computation times for SSAHA and BLAT do not include pre-indexing of the genome (see text).

^b ^Results using the repeat-masked genome are listed first, followed by results from the unmasked genome in parenthesis.

In contrast to the near-perfect recall exhibited by the localization programs, the precision of the programs suffers from a substantial incidence of false positives (Figure 1A). At the genic level, 46% of all reported full-length gene localizations and 16% of sequence tag localizations by SSAHA do not overlap with the known gene localization. For MegaBLAST, 43% of reported gene localizations and 15% of sequence tag localizations are false positives. BLAT shows similar performance, with 38% of reported gene localizations and 15% of sequence tag localizations falling outside the region of the known gene. Generally, the false positives score significantly lower than the true positives.

False positives at the level of the gene may not be problematic, however, since the most common method of interpreting localization results is to accept the highest-scoring match as correct rather than analyzing all returned matches. Correct localizations generally exhibit long, high percent-identity matches, which contribute to higher scores compared with incorrect matches, which are generally short or contain mismatches. The strategy of taking the top hit is largely successful with both full-length gene queries and sequence tag queries (Figure 1A). The SSAHA localization with the highest score is almost always correct, as it overlaps with the known localization of a gene for 99% of full-length gene queries and 98% of sequence tag queries. The MegaBLAST localization with the highest score is correct for 93% of full-length gene queries, and 95% of sequence tag queries. The BLAT localization with the highest score is correct for 99% of full-length gene queries and 99% of sequence tag queries.

Erroneous matches are also less likely to group together on a chromosome than correct matches, which track with exon ordering. While all three programs report matches grouped by chromosome, only the BLAT algorithm incorporates matches in close proximity into a single multi-part alignment, which is given a score that combines the scores of the individual matches in the alignment. This ensures that the top-scoring match is a composite of all matches likely to be exons of the same gene. Another consequence of this grouping is that the scores of correct and incorrect matches are more widely separated than with SSAHA or MegaBLAST.

### Pseudogenes

**Figure 2:**
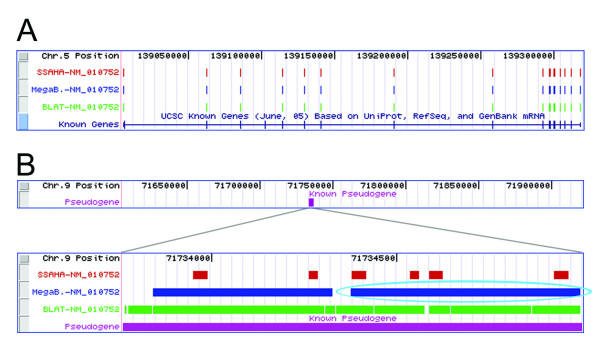
**An example of localization to a pseudogene**. Localization results for the full-length gene encoding mitotic arrest deficient 1-like 1 (Mad1l1), GenBank accession NM_010752. All representations of alignments between query sequences and build 34 of the mouse genome were made using the UCSC Genome Browser Custom Tracks feature. Slight alterations have been made to the representations, including the removal of graphical elements to improve the clarity of the figure, but no changes were made to the alignments. (A) The coordinates of the known gene on the genome are listed at the top, and positions of exons are represented by colored blocks. A region of chromosome 5 is shown containing the known localization of NM_010752 (the Known Genes track at bottom) and the alignments of exons for NM_010752 to the genome by SSAHA, MegaBLAST, and BLAT. (B) A region of chromosome 9 containing a pseudogene related to NM_010752 is shown on the same scale as (A). Below this, the segment of chromosome 9 containing the pseudogene is enlarged. The highest-scoring MegaBLAST match, circled in cyan, localizes to this pseudogene rather than the real gene. The highest scoring matches returned by SSAHA and BLAT are located on chromosome 5 and overlap with the correct localization.

The presence of pseudogenes can confound rules for separating correct from incorrect matches at the genic level for both full-length genes and sequence tags. Pseudogenes are regions of the genome that are very similar in sequence to known genes, but are usually rendered non-functional by mutations or missing elements that prevent transcription or translation. About 80% of pseudogenes are processed pseudogenes, which resemble partial or full-length mRNA sequences that have been integrated into the genome [18]. These are caused by the retrotransposition of double-stranded DNA, read off of single-stranded RNA, into the genome. As processed pseudogenes lack introns, alignments can be constructed between pseudogenes and query sequences that are longer than individual exons. Such alignments may be sufficiently long that penalties accrued for mismatches are more than offset by this longer match length, allowing them to outscore correct matches to exons. In the case of our sequence tags, these alignments are invariably incorrect, since with our method of gene trapping, disruption of a gene is only detected when the vector is inserted into an intron [1]. Figure 2 gives an example that illustrates the difficulty in distinguishing localization to a processed pseudogene from localization to a true gene. More rarely, pseudogenes can be caused by duplications of chromosome segments. These unprocessed pseudogenes contain introns and are therefore less likely to result in high-scoring (but incorrect) matches based on alignment length alone. In addition, a recent duplication can result in a pseudogene with so few mutations that it may be difficult to distinguish it from the coding gene. Although it is possible for a gene-trapping vector to insert into an unprocessed pseudogene containing introns, none were detected in our data set, and thus all localizations to pseudogenes were considered false positives.

As shown in Figure 2, genic localization is compromised by the presence of pseudogenes to varying degrees. SSAHA identifies only exact matches, rather than very similar matches, lending the algorithm a distinct advantage in terms of distinguishing correct matches from pseudogene matches. BLAT alignments can contain mismatches accrued during the alignment extension stage, which increases the likelihood of a high-scoring match to a pseudogene. However, the BLAT score reflects all matches in a region of the genome so that short perfect or near-perfect exon matches in aggregate are likely to outscore longer imperfect matches to pseudogenes. MegaBLAST is the most susceptible to pseudogene matches, as it is relatively tolerant of mismatches and does not have a mechanism for favoring short perfect matches over long imperfect ones.

In this study, pseudogenes may have been the cause of over 100 top-scoring matches that are incorrect, despite high sequence identity between the query sequences and the genome. It is difficult to determine the exact number of incorrect localizations to pseudogenes as relatively few mouse pseudogenes have been annotated. As many as 4000 mouse pseudogenes are predicted to exist [19], and in the closely related human genome, a careful study of an early build of chromosome 22 revealed that 19% of sequences defined as coding likely belong to pseudogenes instead [20]. The distribution of pseudogene matches among the programs varies as might be expected from their algorithmic differences. SSAHA reports a top-scoring match to a region annotated as a probable pseudogene for 17 full-length genes and 60 sequence tags, while BLAT incorrectly localizes 7 genes and 45 sequence tags to probable pseudogenes. MegaBLAST reports top-scoring matches to probable pseudogenes for 116 genes and 162 sequence tags.

### Localization to the correct exon

With respect to recall, all three algorithms perform similarly well in localizing query sequences to the exons of their corresponding genes. For full-length gene queries, SSAHA, MegaBLAST, and BLAT all have exon recall of about 99% (Figure 1B). The sequence tags used in this study are generally substantially shorter than the full-length genes, averaging 255 nucleotides in length, versus 3611 nucleotides for genes, and it is rare that all exons of a gene will be matched in a sequence tag alignment. Thus, exon and nucleotide recall for sequence tag queries should be viewed in a comparative manner, rather than as a direct measure of the accuracy of each algorithm. SSAHA detects 22% of control exons, MegaBLAST detects 22% of control exons, and BLAT detects 23% of control exons.

**Figure 3:**
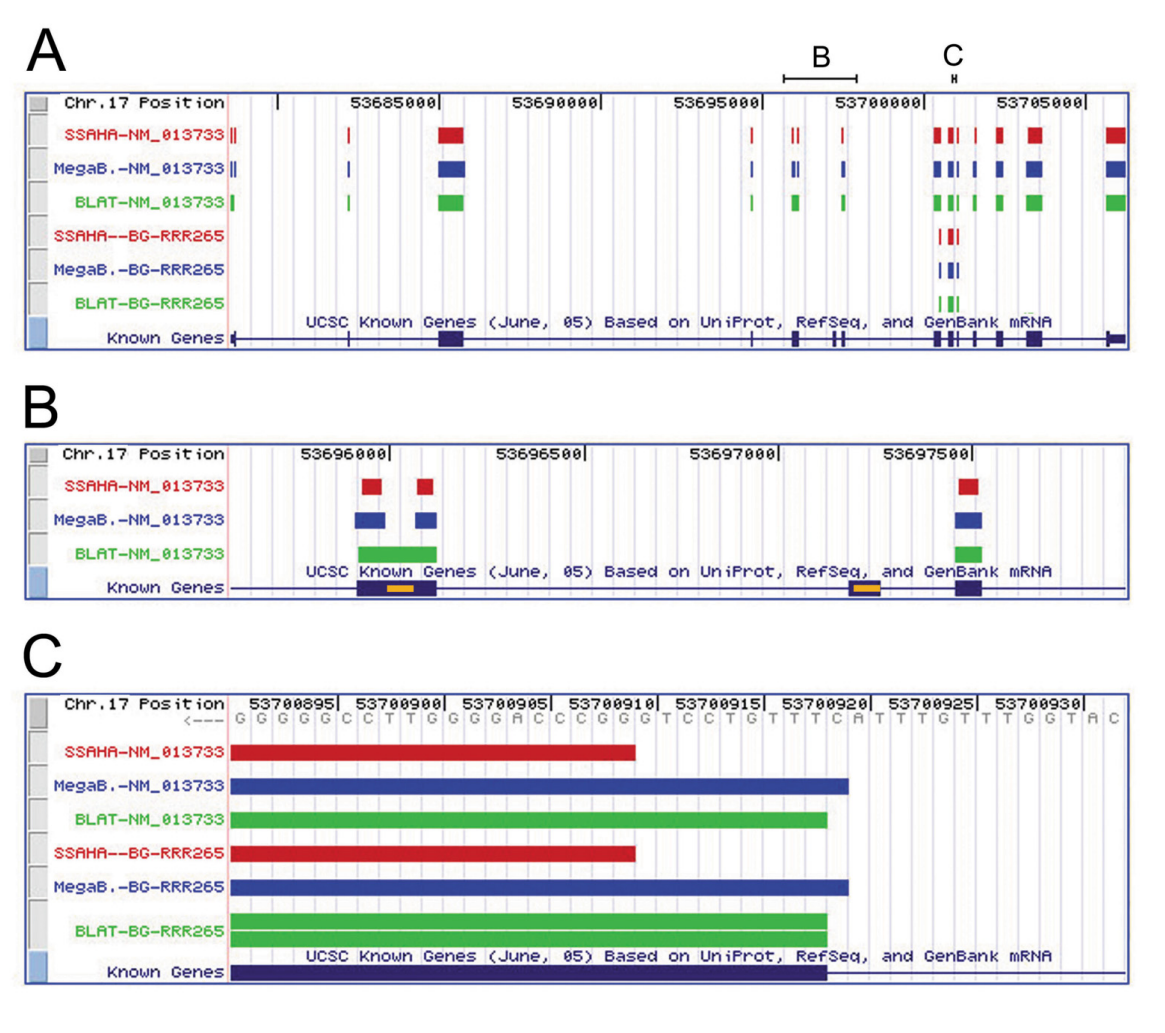
**A representative genome alignment of a full-length gene and a sequence tag**. The full-length gene encoding chromatin assembly factor 1, subunit A (Chaf1a), NCBI accession NM_013733, and the sequence tag BG-RRR265 align to a region of chromosome 17. (A) Overview showing the full region of the genome spanned by Chaf1a. Segments enlarged in the parts B-C are marked above the genome position. (B) Regions of genome that have been removed from the search space by repeat masking are shown in yellow, superimposed on the known gene track. The removal of these regions prevents correct localization of the full-length gene and sequence tag for these exons. (C) Magnification of the exon from region C illustrates differences between the alignment programs in aligning sequence to the edges of exons.

Many of the exons that are not detected overlap with regions of the genome removed from the search space by repeat masking. Two examples of the effect of repeat masking on exon localization are illustrated in Figure 3, which depicts the genome alignment of the full-length gene encoding chromatin assembly factor 1, subunit A (Chaf1a), NCBI accession NM_013733, and the sequence tag BG-RRR265. Each program localizes NM_013733 to the left-most exon shown in Figure 3B by detecting perfect matches on either side of the repeat mask region. BLAT connects these matches because its default parameter settings allow alignments adjacent to a masked region to be extended into the masked sequence while SSAHA and MegaBLAST, whose default settings do not allow alignment to masked regions (see Methods), show the masked region as a gap. The middle exon in Figure 3B does not contain enough unmasked sequence for any algorithm to seed a match, and is thus entirely undetected.

Precision in exon localization is similar for full-length genes and sequence tags, despite the discrepancy in the number of exons matched by the two query types (Figure 1B). This indicates that although the short sequence tags do not contain regions matching all exons of their cognate genes, those with adequate length to be associated with a unique transcript generally contain sufficient information to be localized with high precision. For genes, 78% of SSAHA localization results overlap with known exons, compared with 85% for sequence tags. MegaBLAST has exon precision of 86% for genes, and 87% for sequence tags. BLAT has exon precision of 75% for genes, and 76% for sequence tags. Very rarely, the coding region of a gene contains an intron so short that MegaBLAST will align through it, including the intron in the alignment. This results in errors for four genes in the control set which contain introns of either 9 or 12 nucleotides in the upstream untranslated region. As a source of error, this had only a minimal effect on the overall precision for MegaBLAST and had no effect on the results for BLAT and SSAHA.

An interesting result that is not reflected by measures of recall and precision is that each program occasionally returns multiple correct localizations to the same exon. The full-length genes used in this analysis average 12.9 exons, but each program averages more than 13 correct localizations per gene. SSAHA returns 19.5 localizations per gene, with each localization corresponding to an exon or a false positive. On average, 15.2 of these aligned segments overlap with 12.8 exons. MegaBLAST returns 15.6 localizations per gene, with 13.5 of them correctly identifying 12.8 exons. BLAT returns 20.8 localizations per gene, with of them 15.6 correctly identifying 12.9 exons. Multiple localizations to a single exon can occur because masking or mismatches within exons can split what should be one long matched segment into two or more smaller alignments. In addition, BLAT can generate more than one localization to the exact same region of the genome, as illustrated in Figure 3C. This is a known idiosyncrasy of the BLAT program, and is resolved at the UCSC genome browser Web site by removing such repeat matches [21]. This problem results in no appreciable increase in exon or gene recall compared to SSAHA and MegaBLAST, and also no great loss in precision, as most duplications appear to provide a correct localization (Figure 1). Although we cannot ascertain with certainty how many exons and partial exons each sequence tag spans, we expect that they too generate multiple localizations to a single exon.

### Localization to the correct nucleotide

As expected, the greatest variation in the localization results reported by the three programs is at the nucleotide level (Figure 1C). Recall is diminished for SSAHA and MegaBLAST, but remains high for BLAT. SSAHA detects 77% of control nucleotides for gene queries and 7% for sequence tag queries, MegaBLAST detects 89% of control nucleotides for gene queries and 9% for sequence tag queries, and BLAT localizations detect 93% of control nucleotides for gene queries and 9% for sequence tag queries (Figure 1C). Again, recall for sequence tags is so low only because these represent short fragments of genes and so do not contain sufficient information to allow matching a large proportion of the nucleotides comprising the cognate genes.

**Figure 4:**
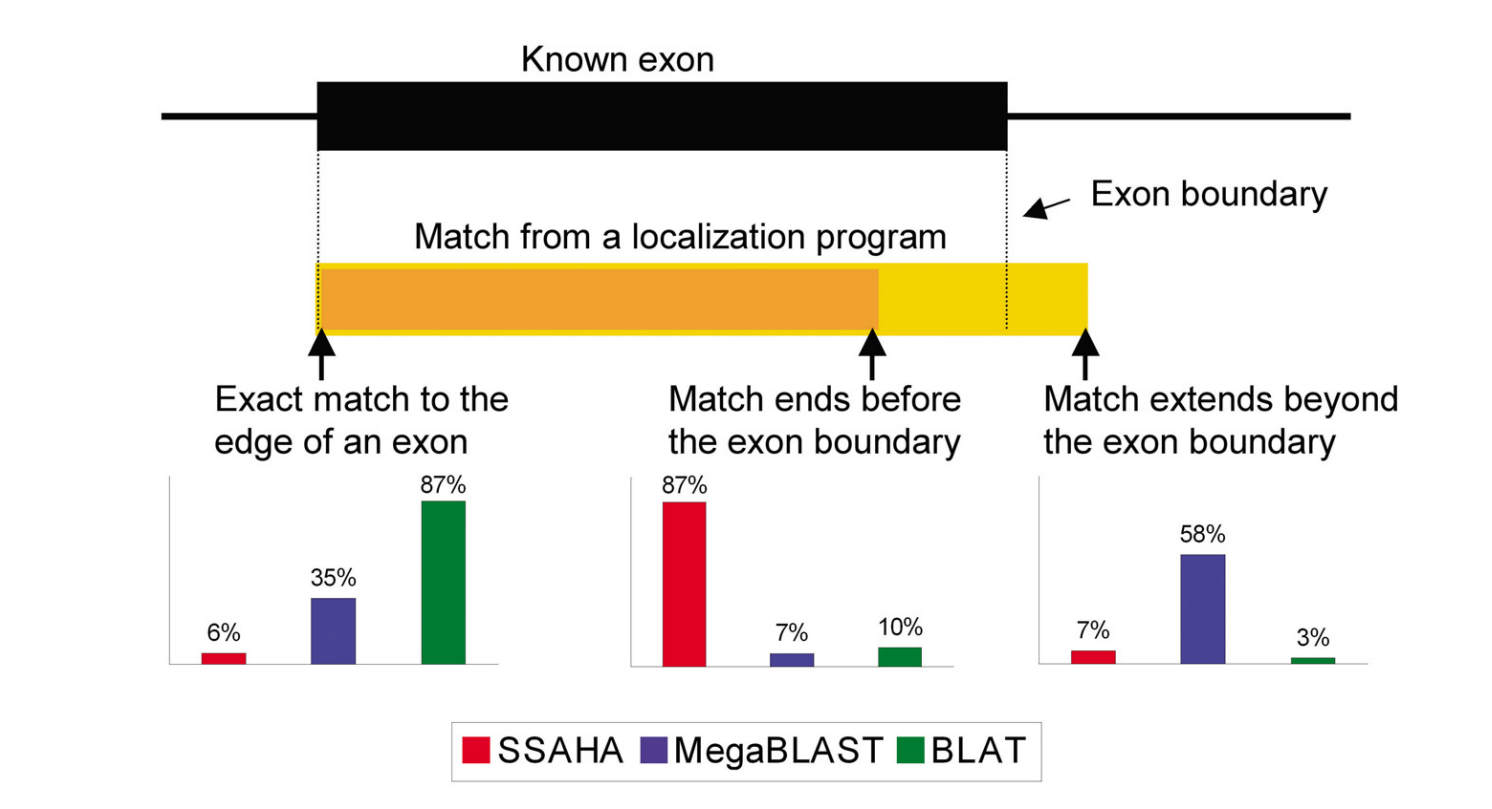
**A summary of the alignments by each program to the edges of exons**. A representation of an exon is shown at top, with a representation of the three possible match outcomes below, i.e., an exact match to the exon boundary, a match that ends before the exon boundary, and a match that extends beyond the exon boundary. The percentage of all matches by each program that fall into those categories are depicted as bar graphs. Left: Percentage of matches correctly aligned to either exon boundary. Middle and right: Percentage of matches incorrectly aligned to an exon boundary, with the match ending before or extending beyond a boundary, respectively.

Diminished recall at the level of individual nucleotides reflects several types of problems, including failure to match to very short exons, misalignment over gaps, and errors in either the query or the genome sequence. The principal cause, however, is difficulty in aligning sequence, using either genes or sequence tags as queries, at the edges of exons. Although failure to accurately align a query to genomic sequence at the edges of exons only slightly lowers the recall levels for each program, each of the three algorithms compared in this study exhibits characteristic problems in localization at the edges of exons, as illustrated in Figure 3C. Figure 4 provides a summary of the performance of each algorithm in exactly matching exon boundaries.

SSAHA correctly matches only 6% of exon boundaries, and only 0.5% of exons (98 of 21,464 total exons) are perfectly matched at both exon edges. The reason for this is that the algorithm splits the genome into non-overlapping fragments that may or may not correlate with exon boundaries. If the edge of an exon does not overlap with an indexed fragment of the genome with a length sufficient to meet the threshold for reporting a match, that fragment will not be included in the match that is returned. Thus, in Figure 3C, SSAHA fails to align 9 nucleotides of both the full-length gene and the sequence tag BG-RRR265 at the 3' edge of the exon because the match does not meet the minimum length of 10 nucleotides. Similarly, small gaps or mismatches that often occur at the ends of sequence tags can interrupt a match, resulting in a minimum loss of 10 nucleotides in the match alignment. (The developers of SSAHA have implemented a new version, SSAHA2 [22], which combines the original SSAHA searching algorithm with a more sensitive alignment program. The changes incorporated in the new version make it likely that SSAHA2 will behave differently from SSAHA. Additionally, associated programs, such as ssahaEST, combine the search and alignment stages of SSAHA2 with several splice site models to increase detection of exon boundaries. SSAHA2 and its associated programs were not included in this analysis as benchmarking and full documentation has not yet been published, although binaries are now available for download from Ensembl.)

In contrast to SSAHA, MegaBLAST often extends alignments beyond the edges of exons. MegaBLAST localizations align up to, but not beyond, exon boundaries in 35% of attempts, with only 11% of exons receiving perfect alignments at both edges. Moreover, the algorithm generates the longest alignments possible, making no attempt to ensure that each nucleotide in the query sequence is matched only once. Thus, MegaBLAST may extend a match beyond the edge of an exon whenever the adjacent intronic sequence coincidentally matches the query sequence (Figure 3C).

BLAT localizations are the most likely to correctly match exon edges, due to the extra steps the algorithm takes to compute correct exon splice sites and match each nucleotide in the query sequence only once. BLAT localizations match exon edges in 87% of attempts, with 79% of exons perfectly aligned at both edges. It is possible that these rates are slightly inflated by counting multiple overlapping correct localizations that occurred in our automated analysis (see Figure 3C for an example). Even so, BLAT has a clear advantage over SSAHA and MegaBLAST in regard to correct identification of exon boundaries.

With respect to precision at the nucleotide level, SSAHA performs the best, achieving the correct localization 92% of the time for both genes and sequence tags. Precision for MegaBLAST and BLAT is also high, with 85% of both sequence tag and full-length gene localizations matching control nucleotides.

### Algorithm speed

Computation times were collected for each localization run. All sequence tags or full-length genes were passed to the localization program as a single file in Fasta format [23] (Table 1). Localizations performed with the unmasked genome were not used in the preceding analysis as this had generally only a small negative effect on recall, but had a large negative impact on precision and analysis speed. (See Figure 3B and associated text for an exception.) SSAHA was the fastest program by about five-fold. MegaBLAST and BLAT were comparable in speed, with BLAT showing an advantage in aligning longer sequences, and MegaBLAST performing more quickly with shorter sequences. Not shown in the Table is the time required for genome indexing, required by both SSAHA and BLAT. This step requires 895.5 seconds for SSAHA, and 399.1 seconds for BLAT, but needs only be run once per genome build.

## Conclusion

Overall, analysis of stand-alone versions of the three localization algorithms, SSAHA, MegaBLAST and BLAT, show that all perform well in localizing both full-length genes and sequence tags to the mouse genome. The differences in performance for sequence tags and full-length reference sequences were surprisingly small, with no program exhibiting significantly diminished performance with sequence tags, despite their generally low quality when compared with full-length reference sequences. While recall and precision performance differ minimally among the programs at the level of gene and exon localization, at a more detailed level, and focusing especially on nucleotide recall, greater variations are found, with different types of characteristic errors associated with each program. Therefore, the choice of the appropriate localization program depends on the specific purpose of the researcher.

As localization to a general region of the genome is performed equally well by all three programs, considerations such as the ease of use of the program and computational speed may become important considerations in choosing which program to use. SSAHA is the fastest program and has the simplest output, so it would seem to be a natural choice for localizing large data sets for general purposes. For automated applications requiring correct localization at the nucleotide level, such as SNP detection or evaluation of alternative splicing, BLAT is currently the best option, as it is distinctly better at aligning the edges of exons. Additionally, the process by which BLAT groups together proximal matches improves the separation between the scores of correct and incorrect matches, increasing confidence in the result. These advantages come at a cost of speed, with BLAT being significantly slower than SSAHA, though comparable in speed with MegaBLAST. For our purpose of localizing gene trap sequence tags to the mouse genome, BLAT was chosen as the program to incorporate into our local annotation pipeline, although use of multiple programs may eventually be implemented to ensure the highest levels of recall and precision.

## Methods

### Sequences

A set of sequence tags for which the localization of the full-length genes are known was used in this study. The sequence tags were derived from knockout experiments performed by members of the IGTC. Initially, 34,138 sequence tags were annotated by using BLAST to search the GenBank non-redundant database [24] for matching gene transcripts. Only those sequence tags that matched a single transcript with at least 95% identity over a contiguous region of at least 90% of the length of the sequence tag, or matched at least 60 contiguous bases at the 3' end of the sequence tag were considered in our analysis. This eliminated the shortest sequence tags, those that matched with multiple genes or genes with multiple differing transcripts, those that matched genes not yet contained in the GenBank non-redundant database, those that did not match a gene, and all sequence tags generated by trapping processes designed to capture introns rather than exons. Additionally, sequence tags were filtered by requiring that their associated gene transcripts be present at each of the major mouse genome browsers, i.e., Ensembl, NCBI, and UCSC. After filtering, a total of 7,043 sequence tags and 3,320 associated gene transcripts remained [see Additional files 2 and 3]. Half of the localizations were not consistent between all genome browsers, leaving a set of 3369 sequence tags associated with 1659 genes all assigned exactly the same coordinates on the mouse genome. The sequence tags range from 32 to 1023 nucleotides in length (mean 255, median 202) and the genes range from 290 to 64,931 nucleotides in length (mean 3611, median 2485).

Sequence tags and their cognate full-length genes were localized in NCBI Mouse Genome Build 34, the fourth major genome build for the mouse [19]. Build 34 is a composite of high quality high-throughput genome sequence and whole-genome shotgun sequence. Localizations were performed with both an unmasked version of the genome and a version with repeat and low-complexity regions removed by RepeatMasker database version 20050112 [17], which uses RepBase update 9.11 [25]. Except as indicated, the results described below were obtained by searching the masked version of the genome, which is the default practice.

### Computation

Alignments were performed on a Hewlett-Packard (HP) AlphaServer GS1280 system, using a single 1.15 GHz processor.

Local versions of online algorithms BLAT, MegaBLAST, and SSAHA were obtained from the genome browser web sites at UCSC, NCBI, and Ensembl, respectively. The most recent versions were chosen, with the exception of BLAT version 26 (February 2004), which was selected because it is the version used to localize BayGenomics sequence tags. SSAHA version 3.1 and MegaBLAST version 2.2.10 represent the most current releases available on July 2005. To approximate the online localization process, parameters were set to match the default parameters employed by the online programs. The three programs do not share the same types of parameters, however, and where the parameters are the same or similar, the values assigned to them are not necessarily consistent. Of particular importance in this study is the default "word length", i.e., the length of indexed genome fragments. A decrease in word length increases the capacity to detect short but real matches, but also increases the number of erroneous matches. Word length was set to 10 nucleotides for SSAHA, and 11 nucleotides for BLAT, with a minimum of two contiguous "words" required to seed a match. Similarly, MegaBLAST requires a minimum of 28 contiguous matches to generate an alignment. How each algorithm deals with repeat masking is also important. None of the algorithms seed alignments in masked regions, but BLAT and MegaBLAST can be set to allow alignments to be extended into regions masked by the RepeatMasker algorithm. By default BLAT is set to allow such alignment extensions, but MegaBLAST is not, resulting in the type of differences between alignments presented in Figure 3B. [see Additional file 4 for a full list of the parameters used for each program.]

A comparison algorithm was devised to demonstrate the accuracy of the localization programs at three levels of granularity relevant for biological inquiry: gene, exon, and nucleotide. At the genic level, any overlap between a localization reported by a program and a known coordinate for a gene was considered a true positive, even if the overlap consisted of a single nucleotide. Similarly, for each exon, only a single nucleotide match was required for a true positive. At the nucleotide level, only an exact match at a single nucleotide position was counted as a true positive. Thus, each level of granularity imposes a different stringency in this analysis. Results are represented by recall and precision scores for each algorithm.

## Authors' contributions

CAH constructed the sequence sets, analyzed the localization data, devised the comparison method and drafted the manuscript. CCH, TEF, and PCB participated in the design of the study. PCB helped to draft the manuscript and conceived of the study. DS installed the localization algorithms, performed the localizations and participated in the design of the study. MK constructed initial test sequence sets. All authors contributed to and approved the final manuscript.

## Supplementary Material

Additional File 1 An example of errors associated with low signal strength in a 5' RACE sequence. (A) Alignment of trace files for the sequence tags BG-XE342 and BG-XH675, both sequenced with 5' RACE, which localize to protein kinase C binding protein 1 (NCBI accession NM_027230). Black arrows indicate the point of vector insertion. The intensity of the signal diminishes towards the 3' end of each sequence. (B) Enlargement of the green-highlighted regions in A. The reverse complement of the trace sequence, which corresponds to the sequence of the inactivated gene, is listed below the expanded trace plots. The low intensity of the signal in this region of the BG-XH675 trace plot results in two nucleotide assignments, circled in pink, that differ from both genomic sequence from chromosome 2 and the associated mRNA sequence for this gene. In contrast, the corresponding nucleotide assignments in the relatively higher quality BG-XE342 trace plot, also circled in pink, agree with the genomic and mRNA sequences. Click here for file

Additional File 2 Sequence tags. A file of sequence tags aligning to known genes that were used in "Comparison of methods for genomic localization of gene trap sequences". This is a smaller set of sequences than is contained in the International Gene Trap Consortium database (). Click here for file

Additional File 3 Genes. A file of full-length genes aligning to the sequence tags that were used in "Comparison of methods for genomic localization of gene trap sequences". Click here for file

Additional File 4 Parameters. A list of parameters used for SSAHA, MegaBlast, and BLAT. Click here for file
