# Supplementary figures and images for: Comparison of methods for genomic localization of gene trap sequences
Source: BMC Genomics. 2006 Sep 18;7:236. doi: 10.1186/1471-2164-7-236 (PMC1617135; doi:10.1186/1471-2164-7-236)

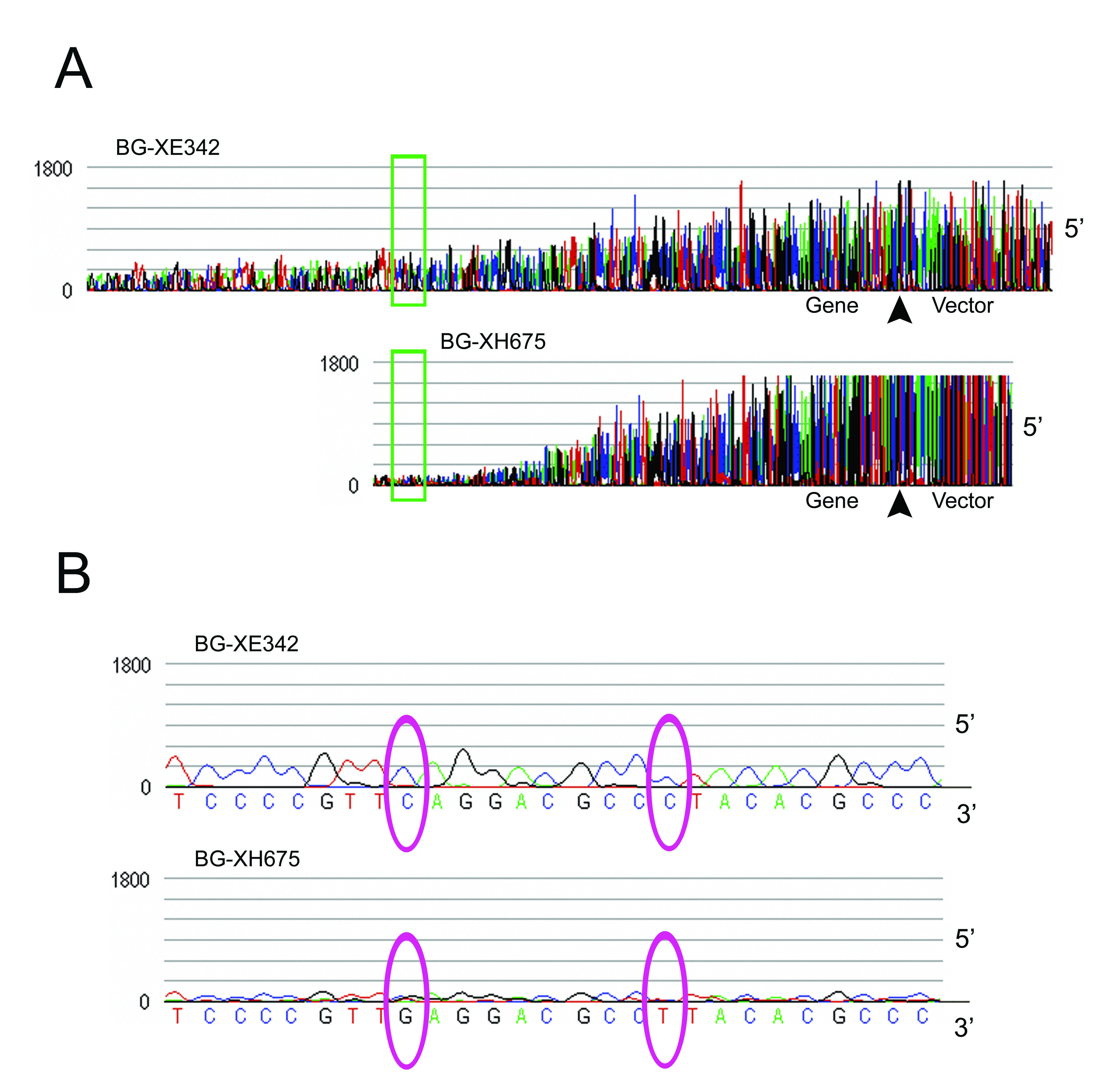

Supplement: Additional File 1 — An example of errors associated with low signal strength in a 5' RACE sequence. (A) Alignment of trace files for the sequence tags BG-XE342 and BG-XH675, both sequenced with 5' RACE, which localize to protein kinase C binding protein 1 (NCBI accession NM_027230). Black arrows indicate the point of vector insertion. The intensity of the signal diminishes towards the 3' end of each sequence. (B) Enlargement of the green-highlighted regions in A. The reverse complement of the trace sequence, which corresponds to the sequence of the inactivated gene, is listed below the expanded trace plots. The low intensity of the signal in this region of the BG-XH675 trace plot results in two nucleotide assignments, circled in pink, that differ from both genomic sequence from chromosome 2 and the associated mRNA sequence for this gene. In contrast, the corresponding nucleotide assignments in the relatively higher quality BG-XE342 trace plot, also circled in pink, agree with the genomic and mRNA sequences. [file 1471-2164-7-236-S1.jpeg]
