# Supplementary material for: Comparison of methods for genomic localization of gene trap sequences
Source: BMC Genomics. 2006 Sep 18;7:236. doi: 10.1186/1471-2164-7-236 (PMC1617135; doi:10.1186/1471-2164-7-236)
Supplement: Additional File 4 — Parameters. A list of parameters used for SSAHA, MegaBlast, and BLAT. [file 1471-2164-7-236-S4.doc]

Parameters for each program used. Descriptions of the parameters have been adapted from the accompanying documentation for the MegaBLAST, SSAHA, and BLAT stand-alone programs.

MegaBLAST parameters

-d Database = goldenpath_ucsc_mouse_masked.fa (mouse genome build 34, Fasta file)

-e Maximum allowed expectation value = 1000000.0 (actual maximum was 0.003)

-m alignment view = tabular

-F Filter query sequence = False

-X X dropoff value for gapped alignment (in bits) = 20

-I Show GI's in deflines = False

-q Penalty for a nucleotide mismatch = -3

-r Reward for a nucleotide match = 1

-v Number of database sequences to show one-line descriptions for = 500

-b Number of database sequence to show alignments for = 0

-D Type of output = tab-delimited one line format

-a Number of processors to use = 1

-M Maximal total length of queries for a single search = 20000000

-W Word size (length of best perfect match) = 28

-z Effective length of the database (use zero for the real size) = 0

-P Maximal number of positions for a hash value (set to 0 to ignore) = 0

-S Query strands to search against database = both

-T Produce HTML output = False

-G Cost to open a gap (zero invokes default behavior) = 0

-E Cost to extend a gap (zero invokes default behavior) = 0

-s Minimal hit score to report (0 for default behavior) = 0

-f Show full IDs in the output (default - only GIs or accessions) = False

-U Use lower case filtering of FASTA sequence = False

-R Report the log information at the end of output = False

-p Identity percentage cut-off = 0

-A Multiple Hits window size = 0

-y X dropoff value for ungapped extension = 10

-Z X dropoff value for dynamic programming gapped extension = 50

-t Length of a discontiguous word template (contiguous word if 0) = 0

-g Generate words for every base of the database (default is every 4th base) = False

-n Use non-greedy (dynamic programming) extension for affine gap scores = False

-N Type of a discontiguous word template = coding

-H Maximal number of HSPs to save per database sequence = unlimited

SSAHA parameters

-queryFormat = fasta file

-subjectFormat = fasta file: goldenpath_ucsc_mouse_masked.fa (mouse genome build 34, Fasta file)

-queryType = DNA

-subjectType = DNA

-parserFriendly = pf Show one match per line as a set of tab delimited fields:

match direction: F forward, R reverse

query name

query start

query end

subject name

subject start

subject end

number of matching bases

percentage identity

-logMode -lm Controls the output of log information

cerr - send to standard error

-packHits -ph Store position of each word in a "packed"

format comprising 32 bits per word. This halves

the size of the .body file at the expense of a

slight decrease in search speed.

-wordLength = 10

-maxGap = 0 Maximum gap allowed between successive hits for

them to count as part of the same match.

-maxInsert = 0 Maximum number of insertions/deletions allowed

between successive hits for them to count as part

of the same match.

-maxStore = 10000 Largest number of times that a word may occur in

the hash table for it to be used for matching

expressed as a multiple of the number of

occurrences per word that would be expected

for a random database of the same size as the

subject database.

-numRepeats = 0 Maximum size of tandem repeating motif that can be

detected in the query sequence. This option may

produce faster and better matches when dealing

with data containing tandem repeats.

-minPrint = 1 The minimum number of matching bases or residues

that must be found in the query and subject

sequences before they are considered as a match

and thus printed.

-queryStart = 1 Specifies the number of the first query sequence to

be matched with the subject sequences (numbering of

both the query and subject sequences starts at 1).

-queryEnd = not specified Specifies the number of the last query sequence to

be matched with the subject sequences. If not

specified, continues until the end of the query

sequence data is reached.

-reverseQuery = yes When matching the reverse strand of a query,

convert the positions of any matches found

into the coordinate frame of the forward strand.

Has no effect if queryType is set to protein.

-sortMatches = 0 Output only the top n matches for each query,

sorted by number of matching bases, then by

subject name, then by start position in the

query sequence.

Default value is zero, which outputs all matches

for each query and does no sorting.

-stepLength = 10 Number of base pairs gap between words used to

produce hash table. Ignored if a precomputed

hash table is being used. Default value is

equal to wordLength.

-queryReplace = default Specifies behaviour upon encountering unexpected

alphanumeric characters in query sequences:

Default: replace with 'A' for DNA, 'X' for protein

-subjectReplace =tag Specifies behaviour upon encountering unexpected

alphanumeric characters in subject sequences:

tag - `tag' the word so that it is not put

into the hash table.

-substituteWords = no Look for single base/amino mismatches in words

that occur less than this many times more often

than would be expected for a random database of

the same size as the subject database.

-bandExtension = 0 Specify size of the band to use for banded dynamic

programming, when producing a graphical alignment.

0 - diagonal only

BLAT parameters

   -t Database type = dna: goldenpath_ucsc_mouse_masked.fa (mouse genome build 34, Fasta file)

   -q Query type =  dna - DNA sequence

   -ooc Use overused tile file = 11.ooc

   -tileSize sets the size of match that triggers an alignment = 11

   -oneOff Mismatches allowed in tile = 0

   -minMatch sets the number of tile matches = 2

   -minScore This is twice the matches minus the mismatches minus some sort of gap penalty = 30

   -minIdentity Sets minimum sequence identity (in percent) = 90

   -maxGap sets the size of maximum gap between tiles in a clump = 2

   -repMatch sets the number of repetitions of a tile allowed before it is marked as overused = 1024

   -minRepDivergence minimum percent divergence of repeats to allow them to be unmasked = 15

   -out output file format = psl (Tab separated format without actual sequence)
